# First in man in-situ augmented reality pedicle screw navigation

**DOI:** 10.1016/j.xnsj.2021.100065

**Published:** 2021-05-01

**Authors:** Mazda Farshad, Philipp Fürnstahl, José Miguel Spirig

**Affiliations:** a Spine Division, Balgrist University Hospital, University of Zurich, Forchstrasse 340, 8008 Zurich, Switzerland; b ROCS: Research in Orthopedic Computer Science, Balgrist University Hospital, University of Zurich, Forchstrasse 340, 8008, Zurich, Switzerland

**Keywords:** Spinal navigation, Augmented reality, Pedicle screw navigation, HoloLens, Case report

## Abstract

**Background:**

Augmented reality (AR) is a rising technology gaining increasing utility in medicine. By superimposing the surgical site and the operator's visual field with computer-generated information, it has the potential to enhance the cognitive skills of surgeons. This is the report of the first in man case with "direct holographic navigation" as part of a randomized controlled trial.

**Case description:**

A pointing instrument was equipped with a sterile fiducial marker, which was used to obtain a digital representation of the intraoperative bony anatomy of the lumbar spine. Subsequently, a previously validated registration method was applied to superimpose the surgery plan with the intraoperative anatomy. The registration result is shown in situ as a 3D AR hologram of the preoperative 3D vertebra model with the planned screw trajectory and entry point for validation and approval by the surgeon. After achieving alignment with the surgery plan, a borehole is drilled and the pedicle screw placed. Postoperativ computer tomography was used to measure accuracy of this novel method for surgical navigation.

**Outcome:**

Correct screw positions entirely within bone were documented with a postoperative CT, with an accuracy similar to current standard of care methods for surgical navigation. The patient was mobilized uneventfully on the first postoperative day with little pain medication and dismissed on the fourth postoperative day.

**Conclusion:**

This first in man report of direct AR navigation demonstrates feasibility in vivo. The continuation of this randomized controlled study will evaluate the value of this novel technology.

## Background

Augmented reality (AR) is a rising technology gaining increasing application in medicine. By superimposing the surgical site and the operator's visual field with computer-generated information, it has the potential to enhance the cognitive skills of surgeons. One crucial task in spine surgery is pedicle screw placement, which bears the risk of neurovascular injury or insufficient screw hold in case of inaccurate screw placement. In order to improve safety and accuracy of screw placement, navigational tools such as optical navigation systems [1], patient-specific instrumentation [2], and even robotic-assisted pedicle screw placement [3] have been developed.

In the last years, substantial efforts have been made to introduce AR as a novel surgical navigation technology into spine surgery [4], [5], [6], [7], [8], [9], [10], [11], [12], [13], [14]. Although promising results have been achieved in feasibility studies, only a few methods demonstrated efficiency in patients [6,14]. The aim of our research was to develop a method capable of visualizing the planned screw trajectories by a computer-generated hologram directly on the real surgical situs, which would enable the surgeon to constantly reconcile the surgical task with the navigation information in an intuitive way.

By leveraging surface digitization and inside-out-tracking, we developed a radiation-free approach for the registration of the preoperative plan to the intraoperative anatomy with only an AR head mounted device (HoloLens 2, Microsoft, Redmond, USA) and a marker-equipped pointer [7]. In this manner, expensive navigation systems with external cameras may be replaced by an affordable surgeon-centered navigation approach, which does not suffer from line-of-sight issues.

After completing pre-clinical validation, the first-in-man randomized controlled trial for AR-based holographic surgical navigation of pedicle screw placement in spine surgery could be started. In the following case description, we report on the case of the first patient treated with “direct” holographic spinal navigation.

## Case description

Approval by the local ethics committees (NCT04610411) and the national agency for therapeutic products (Swissmedic; EUDAMED reference number: 19-02-027424)) for using the technology as a medical device within a clinical trial was obtained.

**Fig. 1:**
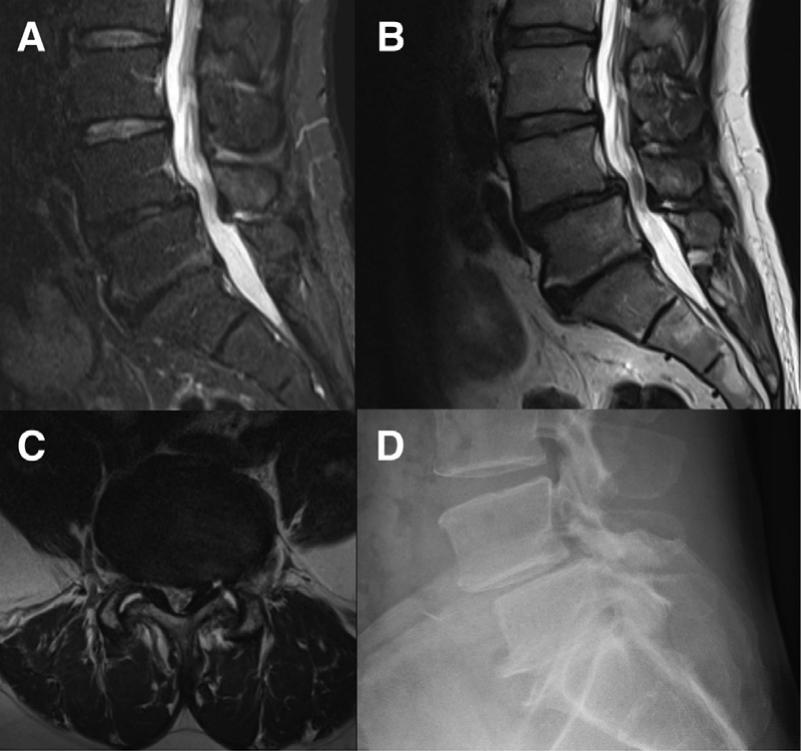
Preoperative images: (A) sagittal fat suppressed MRI (turbo inversion recovery magnitude (TIRM)) and (B) sagittal MRI (T2 sequence) demonstrating segment degeneration at L4/5 and L5/S1, (C) axial MRI (T2 sequence) at level L4/5 showing spinal stenosis, (D) lateral radiograph showing accentuated spondylolisthesis at L4/5 in standing position.

A standard two-level lumbar fusion case was chosen for the first-in-man application on a 57-year-old patient with severe refractory lumbar back and left leg pain due to L5 nerve root radiculopathy. MR and CT images showed degenerative spondylolisthesis at L4/5 with facet joint effusions, consecutive spinal stenosis, and bilateral foraminal stenosis. Advanced degeneration was also detected at the level L5/S1 with almost completely collapsed disc height, intervertebral osteochondrosis (Modic Type 1), and facet joint osteoarthritis. Indication for fusion from L4 to S1 was given (Fig. 1). The patient gave informed consent to be treated with AR-based holographic surgical navigation.

### Surgical planning

**Fig. 2:**
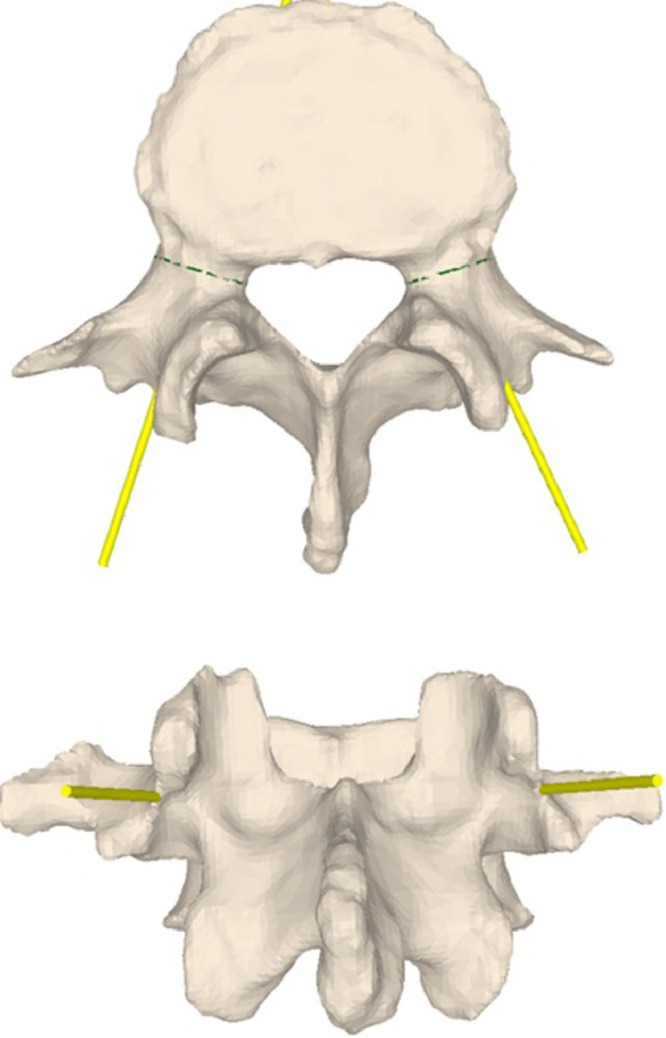
: Preoperative CT reconstructions with planned screw trajectories (yellow).

Preoperative lumbar CT data with a slice thickness of 1 mm (SOMATOM Edge Plus, Siemens Healthcare GmbH, Erlangen, Germany) were acquired, from which a 3D triangular surface model of each vertebra was generated using commercial segmentation software (Mimics 19.0, Materialise NV, Leuven, Belgium). An in-house developed surgical planning software was used to plan pedicles screw insertion points and trajectories in 3D. The screws were visualized as cylindrical primitives, which were manually placed on the 3D vertebra models by a surgeon. The trajectories were planned along the anatomic pedicle axis, with the entry point at the intersection between the transverse process and superior articular facet (Fig. 2). The insertion points and trajectories were then parameterized as 3D locations and direction vectors, and used as navigation information.

### Surgical procedure

**Fig. 3:**
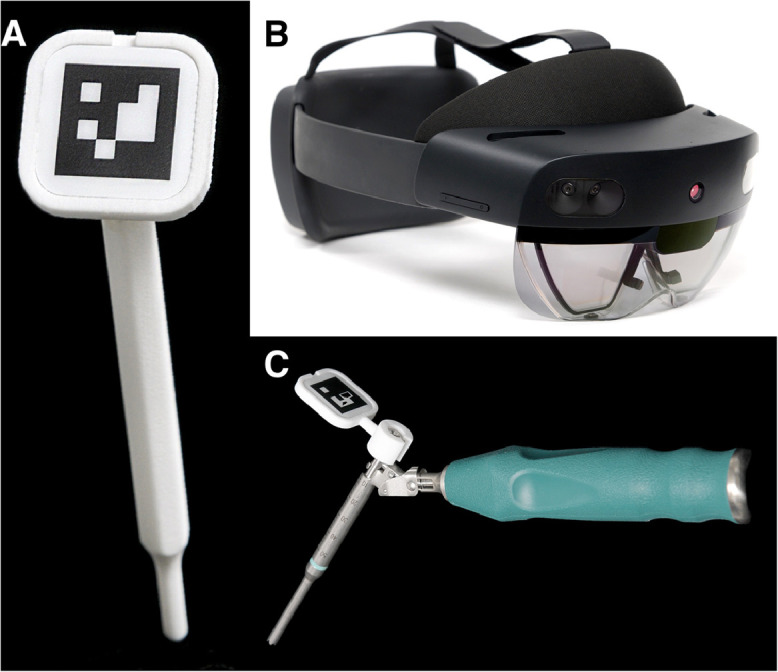
Navigation equipment: (A) 3D printed pointer with fiducial marker, (B) HoloLens 2, (C) drill sleeve guide with fiducial marker mounted on a 3D printed clamp.

The surgical planning data was stored locally on the Hololens device, which was then prepared for surgery following a validated cleaning procedure. A trackable pointer and a clamp for fixation of a marker on a drill sleeve guide were additively manufactured using biocompatible polyamide PA2200 and sterilized in our institution using steam pressure (Fig. 3). The surgical procedure was performed under general anesthesia with the patient in the prone position. The dorsal structures of the spine, such as the spinous process, lamina, and transverse process, were exposed from the midline in a subperiosteal manner as usual.

### Registration of the bony anatomy

**Fig. 4:**
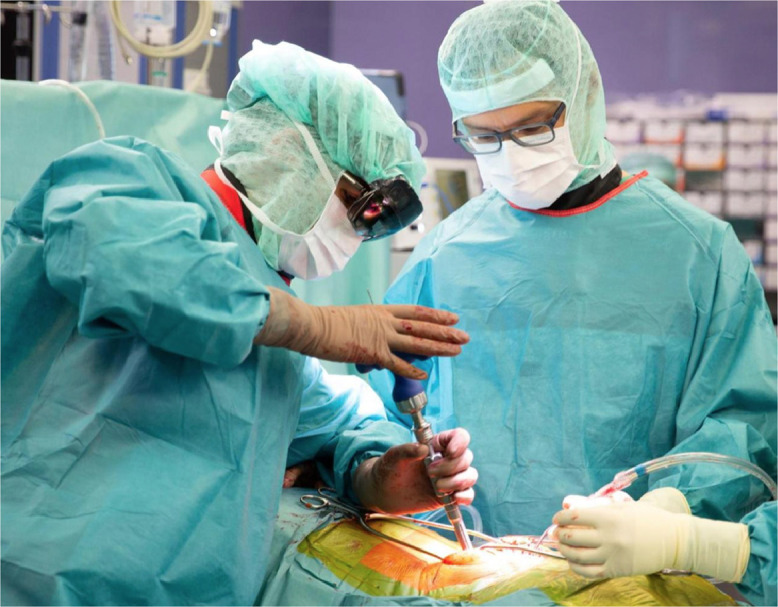
Surgeon with augmented reality head mounted device during navigation.

**Fig. 5:**
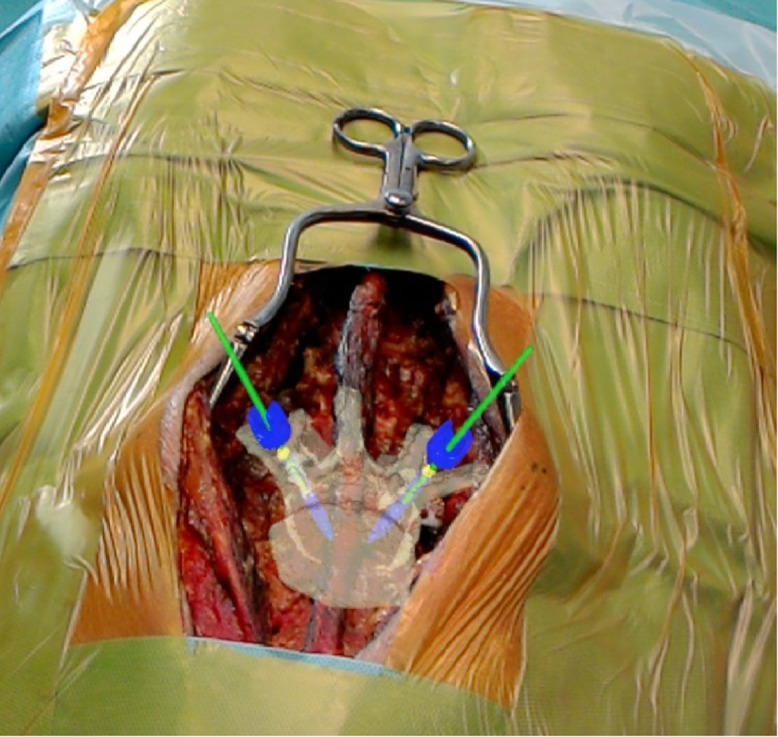
3D hologram of the preoperative 3D vertebra model with the planned screw trajectories projected in situ after registration in order to be validated by the surgeon.

After exposure, the pre-calibrated HoloLens device was placed on the surgeon's head. The surgeon controlled the navigation process with gestures and voice commands (Fig. 4). The pointing instrument was equipped with a sterile fiducial marker (Clear Guide Medical, Baltimore, MD, USA) and used to generate a digital representation of the intraoperative bony anatomy. To this end, the surgeon carefully followed the contours of the spinous process, lamina, and transverse process with the tracked pointer. Marker tracking was implemented using the Aruco library, which was adopted work with Hololens 2. After acquisition of the 3D point cloud of the bony surface, a previously validated and published [7] registration method was applied to superimpose the surgery plan with the intraoperative anatomy. The registration result was presented in-situ as a 3D hologram of the preoperative 3D vertebra model with the planned screw trajectory and entry point for validation and approval (Fig. 5). Registration was done separately for each vertebra.

## Navigation

The L4 and L5 screws were placed using an AR-based holographic surgical navigation without any fluoroscopic control. A conventional drill sleeve guide with a depth limit (∅ 3.2 mm No. 03.614.010, Synapse System, DePuy Synthes, J&J) was turned into an AR-trackable instrument by mounting it to a sterile fiducial marker using a sterile 3D-printed clamp (Fig. 3). The navigation was performed visually based on the drill sleeve's position and orientation, which was acquired in real-time with the HoloLens camera and Aruco marker detection [15]. The current Euclidean distance from the planned entry point and the angular deviation from the planned trajectory was in-situ visualized in millimeters and degrees, respectively.

**Fig. 6:**
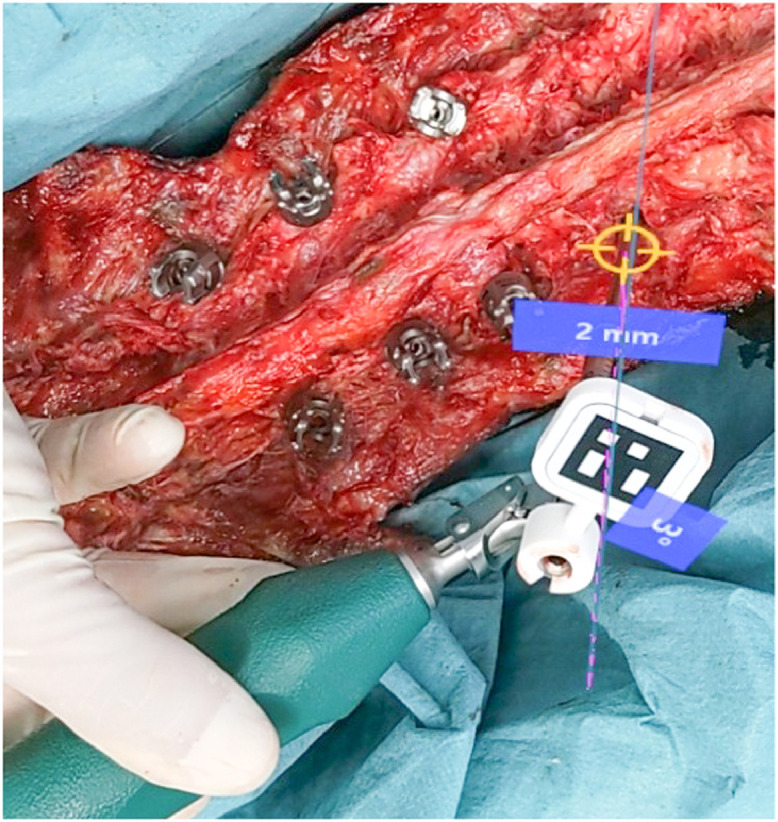
Surgeon's view during navigation showing current deviation of entry point (3 mm) and trajectory (2°) in real time (for safety reasons shown here only in a cadaver sample).

Furthermore, the direction of trajectory deviation was visualized by three points forming a triangle: the first lying on the entry point, the second on the planned trajectory, and the third on the current trajectory (Fig. 6). After achieving alignment with the surgery plan, a borehole was drilled limited to 40 mm depth. The borehole was checked for pedicle wall perforation with a ball tip probe, before inserting blunt k-wires. Finally, cannulated 7 × 45 mm pedicle screws were inserted under K-wire guidance. S1 screws were inserted in a standard manner under anatomic orientation and lateral fluoroscopic control at the end of the screw insertion procedure to limit the experiment to only four screws, as this was a first-in-man procedure. The final screw position was checked by fluoroscopy, showing a satisfying result. Further steps, like decompressive laminotomy and intervertebral cage insertion, were done in a usual manner.

### Outcome

**Fig. 7:**
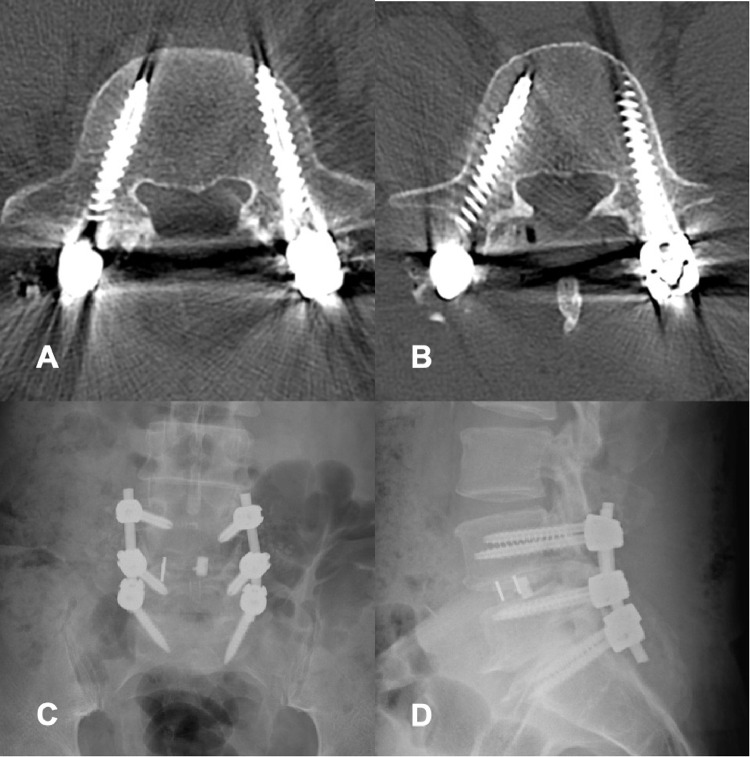
Postoperative images: (A) axial CT at L5 and (B) axial CT at L4 showing adequate position of navigated screws without pedicle perforation, (C) antero-posterior and (D) lateral radiographs showing final spinal fusion construct.

Postoperatively, the patient showed a complete reduction of leg pain and no further signs of radiculopathy. Correct screw positions entirely within bone were documented with a postoperative CT (Fig. 7). The 3D evaluation of the surgical accuracy based on a comparison between preoperative planning and postoperative CT revealed a mean 3D-summed angular deviation of 7.3 ± 3.6° for the trajectories and 3.5 ± 1.9 mm for screw entry points. The patient was mobilized uneventfully on the first postoperative day with little pain medication, and dismissed on the fourth postoperative day.

## Discussion

This is the report of the first-in-man application of a new fluoroscopy-free direct holographic surgical navigation technique with in-situ trajectory guidance. This is an essential step for the implementation of AR as the next-generation surgical navigation in surgery. Although surgical accuracy and user-friendliness have to be investigated with more cases, this case report proves the feasibility of direct holographic surgical navigation in an in-vivo setting.

State-of-the-art navigation technologies in spinal surgery have superior accuracy than the free hand technique [2,[16], [17], [18], [19], [20]]. However, the main limitations of such navigation systems are high set-up and maintenance costs, even if these systems might be cost neutral in the long-term in high volume centers [21,22]. This assumption is supported by a survey by Härtl et al., who revealed that surgeons cited high costs as one of the main reasons for not to use navigation systems [23]. Newer robotic assisted navigation systems are associated with even greater costs [24,25]. From technical viewpoint, a considerable limitation of “traditional” optical navigation systems is the dependence to an external camera system, which make it more difficult to have a clear view to the fiducial markers on the anatomy and surgical tools. According to a recent study, line-of-sight problem occurs multiple times in nearly every navigated neurosurgical procedure [26].

Another known limitation is the attention shift, which occurs when the surgeon is obligated to fix the gaze on a remote screen during navigation [27,28]. The approach presented here overcomes such limitations by combining a small, portable, and affordable device with computer vision software (Fig. 3).

However, the here reported novel method of navigation introduces new limitations: First, the operator needs previous training in order to be able to use the system reliably. In our experience, user-dependency seems to be higher at this stage compared to current standard navigations systems. Second, accuracy is dependent on the quality of the registration process and absence of patient motion. Accuracy is high in cadaveric experimental setting with up to 97.5% (unpublished data), comparable to current computer based navigation techniques (96% [16]) or even robotic assisted navigation (95-98% [17,29,30]) and certainly surpassing the conventional free-hand technique (43% to 86% [16,31,32]).

Compared to other navigation techniques, the here presented method seems advantageous, as the surgeon remains the last instance of quality control: He should be able to notice if the projected hologram is not aligned with the anatomy. Eventually, the currently running RCT will provide quantification of accuracy. Third, another potential limitation for broad clinical usage is the potential inconvenience associated with wearing a head mounted device. Further studies evaluating experience and surgeon's acceptance using this navigation are in progress.

So far, we found only a few clinical studies evaluating similar navigation technologies in humans [6,14]. Elmi-Terander and his group uses a sophisticated AR technology based on a video system with four-cameras, permitting fusion of 3D CT information with live video images of the surgical field [6,[33], [34], [35]]. Charles et al. investigated the same system and confirmed applicability in minimal invasive procedures [36]. However, the system of Elmi-Terander et al. is burdened with some degree of attention shift, since the surgeon is still obligated to fix his gaze on a remote screen for navigation. Molina et al. uses a Food and Drug Administration (FDA) approved AR navigation system with a head mounted device which projects navigation information directly into the operator's retina using a transparent near-eyedisplay [14]. In this way, the surgeon sees a 3D segmentation of the spine, overlaying the anatomy, and all navigation information displayed aside. Their approach is promising in reducing attention shift, but their registration method requires to acquire an intraoperative CT [14]. General application of such systems is limited due to the necessity of additional costly equipment. Therefore, we aim to provide an intraoperative image-free method of registration of anatomy. However, our approach without an anchored marker is yet prone to failure in case of position changes of the patient. We believe however that such an error can be noticed by the operator as an obvious offset of the hologram overlay on anatomy.

## Conclusion

This case report presents the first in man application of a portable, fluoroscopy free AR based in situ navigation system. While this innovation overcomes some important disadvantages of the current navigation system, it introduces new challenges that need a careful incremental improvement process.

Funding Disclosure Statement: No financial funding sources were acquired for this case report. This work is part of «SURGENT», a flagship project of University Medicine Zurich/Hochschulmedizin Zürich.

## Declarations of Competing Interests

The authors declare that they have no known competing financial interests or personal relationships that could have appeared to influence the work reported in this paper.

## Appendix Supplementary materials

Image, application 1

Image, application 2

## References

[1] Gelalis ID, Paschos NK, Pakos EE, Politis AN, Arnaoutoglou CM, Karageorgos AC (2012). Accuracy of pedicle screw placement: a systematic review of prospective in vivo studies comparing free hand, fluoroscopy guidance and navigation techniques. Eur Spine J.

[2] Farshad M, Betz M, Farshad-Amacker NA, Moser M. (2016). Accuracy of patient-specific template-guided vs. free-hand fluoroscopically controlled pedicle screw placement in the thoracic and lumbar spine: a randomized cadaveric study. Eur Spine J.

[3] Li HM, Zhang RJ, Shen CL. (2020). Accuracy of pedicle screw placement and clinical outcomes of robot-assisted technique versus conventional freehand technique in spine surgery from nine randomized controlled trials: a meta-analysis. Spine (Phila Pa 1976).

[4] Dennler C, Jaberg L, Spirig J, Agten C, Götschi T, Fürnstahl P (2020). Augmented reality-based navigation increases precision of pedicle screw insertion. J Orthop Surg Res.

[5] Müller F, Roner S, Liebmann F, Spirig JM, Fürnstahl P, Farshad M. (2020). Augmented reality navigation for spinal pedicle screw instrumentation using intraoperative 3D imaging. Spine J.

[6] Elmi-Terander A, Burström G, Nachabe R, Skulason H, Pedersen K, Fagerlund M (2019). Pedicle screw placement using augmented reality surgical navigation with intraoperative 3D imaging: a first in-human prospective cohort study. Spine (Phila Pa 1976).

[7] Liebmann F, Roner S, von Atzigen M, Scaramuzza D, Sutter R, Snedeker J (2019). Pedicle screw navigation using surface digitization on the Microsoft HoloLens. Int J Comput Assist Radiol Surg.

[8] Molina CA, Theodore N, Ahmed AK, Westbroek EM, Mirovsky Y, Harel R (2019). Augmented reality–assisted pedicle screw insertion: a cadaveric proof-of-concept study. J Neurosurg Spine.

[9] Burström G, Nachabe R, Persson O, Edström E, Elmi Terander A. (2019). Augmented and virtual reality instrument tracking for minimally invasive spine surgery. Spine (Phila Pa 1976).

[10] Ma L, Zhao Z, Chen F, Zhang B, Fu L, Liao H. (2017). Augmented reality surgical navigation with ultrasound-assisted registration for pedicle screw placement: a pilot study. Int J Comput Assist Radiol Surg.

[11] Elmi-Terander A, Burström G, Nachabe R, Skulason H, Pedersen K, Fagerlund M (2019). Pedicle screw placement using augmented reality surgical navigation with intraoperative 3D imaging. Spine (Phila Pa 1976).

[12] Edström E, Burström G, Nachabe R, Gerdhem P, Elmi Terander A. (2019). A novel augmented-reality-based surgical navigation system for spine surgery in a hybrid operating room: design, workflow, and clinical applications. Oper Neurosurg.

[13] Wu JR, Wang ML, Liu KC, Hu MH, Lee PY. (2014). Real-time advanced spinal surgery via visible patient model and augmented reality system. Comput Methods Programs Biomed.

[14] Molina CA, Sciubba DM, Greenberg JK, Khan M, Witham T. (2020). Clinical accuracy, technical precision, and workflow of the first in human use of an augmented-reality head-mounted display stereotactic navigation system for spine surgery. Oper Neurosurg.

[15] Garrido-Jurado S, Muñoz-Salinas R, Madrid-Cuevas FJ, MJ Marín-Jiménez (2014). Automatic generation and detection of highly reliable fiducial markers under occlusion. Pattern Recognit.

[16] Mason A, Paulsen R, Babuska JM, Rajpal S, Burneikiene S, Nelson EL (2014). The accuracy of pedicle screw placement using intraoperative image guidance systems. J Neurosurg Spine.

[17] Van Dijk JD, Van Den Ende RPJ, Stramigioli S, Köchling M, Höss N. (2015). Clinical pedicle screw accuracy and deviation from planning in robot-guided spine surgery: Robot-guided pedicle screw accuracy. Spine (Phila Pa 1976).

[18] Meng X, Guan X, Zhang H, He S. (2016). Computer navigation versus fluoroscopy-guided navigation for thoracic pedicle screw placement: a meta-analysis. Neurosurg Rev.

[19] Du JP, Wang DH, Zhang J, Fan Y, Wu QN, Hao DJ. (2018). Accuracy of pedicle screw insertion among 3 image-guided navigation systems: systematic review and meta-analysis. World Neurosurg.

[20] Benech CA, Perez R, Benech F, Greeley SL, Crawford N, Ledonio C. (2019). Navigated robotic assistance results in improved screw accuracy and positive clinical outcomes: an evaluation of the first 54 cases. J Robot Surg.

[21] Lee YC, Lee R. (2020). Image-guided pedicle screws using intraoperative cone-beam CT and navigation. A cost-effectiveness study. J Clin Neurosci.

[22] Dea N, Fisher CG, Batke J, Strelzow J, Mendelsohn D, Paquette SJ (2016). Economic evaluation comparing intraoperative cone beam CT-based navigation and conventional fluoroscopy for the placement of spinal pedicle screws: a patient-level data cost-effectiveness analysis. Spine J.

[23] Härtl R, Lam KS, Wang J, Korge A, Kandziora F, Audigé L. (2013). Worldwide survey on the use of navigation in spine surgery. World Neurosurg.

[24] Fiani B, Quadri SA, Farooqui M, Cathel A, Berman B, Noel J (2020). Impact of robot-assisted spine surgery on health care quality and neurosurgical economics: a systemic review. Neurosurg Rev.

[25] Malham GM, Wells-Quinn T. (2019). What should my hospital buy next?—Guidelines for the acquisition and application of imaging, navigation, and robotics for spine surgery. J Spine Surg.

[26] Mehbodniya AH, Moghavvemi M, Narayanan V, Waran V. (2019). Frequency and causes of line of sight issues during neurosurgical procedures using optical image-guided systems. World Neurosurg.

[27] Rahmathulla G, Nottmeier EW, Pirris SM, Deen HG, Pichelmann MA. (2014). Intraoperative image-guided spinal navigation: technical pitfalls and their avoidance. Neurosurg Focus.

[28] Léger É, Drouin S, Collins DL, Popa T, Kersten-Oertel M. (2017). Quantifying attention shifts in augmented reality image-guided neurosurgery. Healthc Technol Lett.

[29] Laudato PA, Pierzchala K, Schizas C. (2018). Pedicle screw insertion accuracy using O-arm, robotic guidance, or freehand technique. Spine (Phila Pa 1976).

[30] Zhang JN, Fan Y, Hao DJ. (2019). Risk factors for robot-assisted spinal pedicle screw malposition. Sci Rep.

[31] Chan A, Parent E, Narvacan K, San C, Lou E. (2017). Intraoperative image guidance compared with free-hand methods in adolescent idiopathic scoliosis posterior spinal surgery: a systematic review on screw-related complications and breach rates. Spine J.

[32] Nevzati E, Marbacher S, Soleman J, Perrig WN, Diepers M, Khamis A (2014). Accuracy of pedicle screw placement in the thoracic and lumbosacral spine using a conventional intraoperative fluoroscopy-guided technique: a national neurosurgical education and training center analysis of 1236 consecutive screws. World Neurosurg.

[33] Elmi-Terander A, Burström G, Nachabé R, Fagerlund M, Ståhl F, Charalampidis A (2020). Augmented reality navigation with intraoperative 3D imaging vs fluoroscopy-assisted free-hand surgery for spine fixation surgery: a matched-control study comparing accuracy. Sci Rep.

[34] Edström E, Burström G, Persson O, Charalampidis A, Nachabe R, Gerdhem P (2020). Does augmented reality navigation increase pedicle screw density compared to free-hand technique in deformity surgery? single surgeon case series of 44 patients. Spine (Phila Pa 1976).

[35] Edström E, Burström G, Nachabe R, Gerdhem P, Terander AE. (2020). A novel augmented-reality-based surgical navigation system for spine surgery in a hybrid operating room: Design, workflow, and clinical applications. Oper Neurosurg.

[36] Charles YP, Cazzato RL, Nachabe R, Chatterjea A, Steib J-P, Gangi A. (2021). Minimally Invasive transforaminal lumbar interbody fusion using augmented reality surgical navigation for percutaneous pedicle screw placement. Clin Spine Surg.

