# Supplementary material for: First in man in-situ augmented reality pedicle screw navigation
Source: N Am Spine Soc J. 2021 May 1;6:100065. doi: 10.1016/j.xnsj.2021.100065 (PMC8819976; doi:10.1016/j.xnsj.2021.100065)
Supplement: Supplementary file 1 [file mmc1.pdf]

THE SPINE JOURNAL

A Multidisciplinary Journal of Spinal Disorders
Official Journal of the North American Spine Society

FDA APPROVAL STATUS

Article Title: First in man in-situ Augmented Reality pedicle screw navigation

Manuscript reference #:

FDA Approval Status
If a device or drug requiring FDA approval is identified as an important component of your article, you must indicate the FDA status for use as it will be discussed. Please list the name of the device(s) and drug(s) requiring FDA approval and check the appropriate status for use as it is discussed in the article.

☒ My manuscript does not discuss any drugs or devices requiring FDA approval.

1. Device/Drug

◯ Not Applicable ◯ Not approved for this indication ◯ Approved ◯ Investigational

2. Device/Drug

◯ Not Applicable ◯ Not approved for this indication ◯ Approved ◯ Investigational

3. Device/Drug

◯ Not Applicable ◯ Not approved for this indication ◯ Approved ◯ Investigational

4. Device/Drug

◯ Not Applicable ◯ Not approved for this indication ◯ Approved ◯ Investigational

5. Device/Drug

◯ Not Applicable ◯ Not approved for this indication ◯ Approved ◯ Investigational

Corresponding Author Name: Mazda Farshad Date: Jan 17, 2021
