# Supplementary material for: First in man in-situ augmented reality pedicle screw navigation
Source: N Am Spine Soc J. 2021 May 1;6:100065. doi: 10.1016/j.xnsj.2021.100065 (PMC8819976; doi:10.1016/j.xnsj.2021.100065)
Supplement: Supplementary file 2 [file mmc2.pdf]

Balgrist
Universitätsklinik

**Universitätsklinik Balgrist**

# Einverständniserklärung

**Herr Roland F. Schürpf, geb 26.7.1963,**

**erklärt sich mit seiner Unterschrift einverstanden, dass**

**die in Zusammenhang mit der Behandlung vom 1.12.20 in der Universitätsklinik Balgrist gemachten Foto- bzw. Video-Aufnahmen sowie die von ihm ev. gemachten Aussagen im Interview (bei Reportagen) verwendet werden dürfen für**

| | |
|---|---|
| x (Online-) Artikel zum Thema | Augmentierte Realität in der Chirurgie |
| x TV-/Radio-Sendung/Kinofilm | Augmentierte Realität in der Chirurgie |
| x Foto-Reportage zum Thema | Augmentierte Realität in der Chirurgie |
| X Printprodukt (Broschüre, Magazin etc.) | Augmentierte Realität in der Chirurgie |
| x Webseite (Intranet / Internet) | Augmentierte Realität in der Chirurgie |
| x auf Social Media-Kanälen (Facebook, Twitter etc.) | Augmentierte Realität in der Chirurgie |

Zudem wurde Herr Schürpf persönlich von Prof. Farshad am 27.11.20 informiert, dass er der erste Patient in der Studie " Evaluation der Genauigkeit von chirurgischer Navigation bei Instrumentation der Wirbelsäule mittels Augmented Reality („erweiterte Realität")". Dazu wurde eine separate Einwilligungserklärung unterschrieben. Die Technik der Augmentierten Realität wird als Navigationshilfe in dieser Art weltweit das erste Mal eingesetzt.

Ort/Datum ↑

**Herr Roland F. Schürpf, geb 26.7.1963,**

Zürich, 27.11.20

Ort/Datum

Aufklärende Person
Prof. M. Farshad



Universität Zürich UZH | swiss olympic MEDICAL CENTER
